# Optical properties and electrical transport of thin films of terbium(III) bis(phthalocyanine) on cobalt

**DOI:** 10.3762/bjnano.5.215

**Published:** 2014-11-11

**Authors:** Peter Robaschik, Pablo F Siles, Daniel Bülz, Peter Richter, Manuel Monecke, Michael Fronk, Svetlana Klyatskaya, Daniel Grimm, Oliver G Schmidt, Mario Ruben, Dietrich R T Zahn, Georgeta Salvan

**Affiliations:** 1 Semiconductor Physics, Technische Universität Chemnitz, Reichenhainer Straße 70, 09107 Chemnitz, Germany; 2 Material Systems for Nanoelectronics, Technische Universität Chemnitz, Reichenhainer Straße 70, 09107 Chemnitz, Germany; 3 Institute for Integrative Nanosciences, IFW Dresden, Helmholtzstraße 20, 01069 Dresden, Germany; 4 Institute of Nanotechnology, Karlsruhe Institute of Technology (KIT), 76344 Eggenstein-Leopoldshafen, Germany,; 5 Université de Strasbourg, Institut de Physique et de Chimie des Materiaux de Strasbourg, CNRS UMP 7504, 23 Rue du Loess, 67034 Strasbourg Cedex 2, France

**Keywords:** current sensing AFM, ellipsometry, spintronics, TbPc_2_, transport properties

## Abstract

The optical and electrical properties of terbium(III) bis(phthalocyanine) (TbPc_2_) films on cobalt substrates were studied using variable angle spectroscopic ellipsometry (VASE) and current sensing atomic force microscopy (cs-AFM). Thin films of TbPc_2_ with a thickness between 18 nm and 87 nm were prepared by organic molecular beam deposition onto a cobalt layer grown by electron beam evaporation. The molecular orientation of the molecules on the metallic film was estimated from the analysis of the spectroscopic ellipsometry data. A detailed analysis of the AFM topography shows that the TbPc_2_ films consist of islands which increase in size with the thickness of the organic film. Furthermore, the cs-AFM technique allows local variations of the organic film topography to be correlated with electrical transport properties. Local current mapping as well as local I–V spectroscopy shows that despite the granular structure of the films, the electrical transport is uniform through the organic films on the microscale. The AFM-based electrical measurements allow the local charge carrier mobility of the TbPc_2_ thin films to be quantified with nanoscale resolution.

## Introduction

**Figure 1:**
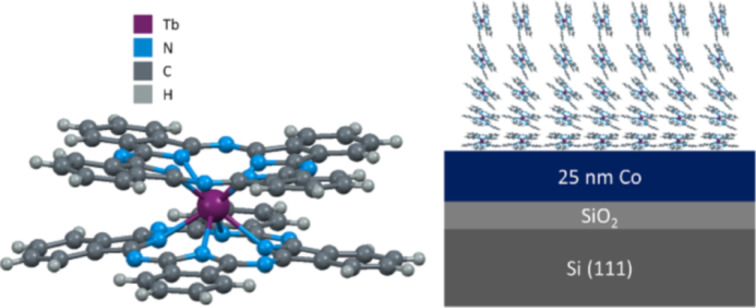
TbPc_2_ molecule (left). Investigated layer stack: TbPc_2_ thin films on cobalt grown on SiO_2_/Si(111).

Molecular spintronic devices could bring a new era of information technology, as the materials are inexpensive and have a potentially higher efficiency than conventional electronic devices [1–9]. Therefore, many studies were carried out to identify organic molecules with suitable properties for spintronics over the past few years. Terbium(III) bis(phthalocyanine) (TbPc_2_) is an excellent candidate to provide all the necessary features for molecular spintronics, as it is both an organic semiconductor and a single molecule magnet (SMM). TbPc_2_ was previously implemented in an organic field effect transistor (OFET) as a hole transporting layer [10] and recently Urdampilleta et al. reported a supramolecular spin valve made of a carbon nanotube (CNT) covered by only a few TbPc_2_ molecules [5]. For electronic and spintronic devices it is crucial to know and to control the molecular orientation on the device-related substrates. The TbPc_2_/Co heterojunction was already proposed to serve as a model system for a SMM semiconducting layer on top of a ferromagnetic electrode for a future spintronic device. The chemical and magnetic properties of this interface were investigated by Klar et al. and it was found that the magnetic moment of the Tb couples antiferromagnetically to the Co substrate [11]. In this work we focus on the study of other device-relevant aspects: the influence of the film thickness, morphology, and molecular orientation on the electrical transport in TbPc_2_ layers on polycrystalline cobalt films. The TbPc_2_ molecule and the investigated layer stack are schematically shown in Figure 1, which includes a sketch of the molecular orientation which will be discussed later. The present manuscript reveals the optical and electrical properties of TbPc_2_ films with different thicknesses on Co substrates as well as the molecular tilt angle and grain size distribution of the samples using spectroscopic ellipsometry, AC atomic force microscopy, and current sensing atomic force microscopy. Topographic and electrical AFM techniques provide a reliable method to investigate and correlate the structural and local electrical properties of TbPc_2_ thin films. This knowledge is crucial for the implementation and fabrication of TbPc_2_-based devices.

## Results and Discussion

### Spectroscopic ellipsometry

**Figure 2:**
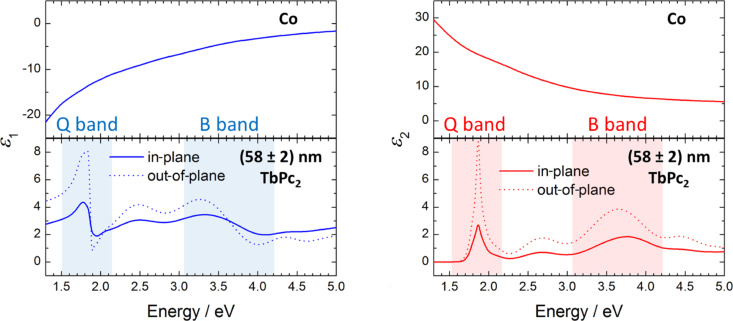
Dielectric function of a TbPc_2_ film on cobalt. The blue lines and the red lines represent the real part (left) and the imaginary part (right), respectively, of the dielectric function. The top graphs show the dielectric function of Co and bottom graphs are obtained from 58 nm TbPc_2_ on top of the Co layer.

For the ellipsometric analysis, four TbPc_2_ films with different thicknesses (18 ± 1 nm, 41 ± 1 nm, 58 ± 2 nm, and 87 ± 3 nm) were prepared by organic molecular beam deposition. The underlying 25 nm thick cobalt film was prepared by electron beam evaporation. Both depositions were performed without breaking the vacuum in between to avoid any oxidation of the Co films. Figure 2 shows the dielectric function, 
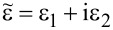
, of Co taken from [12] and a 58 nm TbPc_2_ film on Co. TbPc_2_ films exhibit a uniaxial anisotropy of the dielectric function similar to many planar phthalocyanines, for example, CuPc [13] or H_2_Pc [14]. Consequently, the dielectric function parallel (in-plane) and perpendicular (out-of-plane) to the sample surface differs 
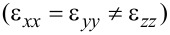
. For a numerical analysis of the measured spectroscopic ellipsometry data a model employing eleven Gaussian oscillators was used. The energy position and the full width half maximum (FWHM) of the oscillators were fixed for all samples, while their amplitudes were allowed to vary during the Kramers–Kronig consistent fitting procedure (experimental data and model fit for one sample: Supporting Information File 1, Figure S1). From the numerical analysis we can extract the anisotropic dielectric function as well as the thickness of the different layers in the stack.

The most prominent absorption bands, namely the Q and B band, of phthalocyanines are highlighted in Figure 2. They correspond to ligand-related π–π* transitions [15].

For organic semiconductors we can consider the relative magnetic permeability to be µ_r_ ≈ 1. Thus, we can easily determine the optical constants 
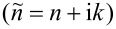
 from the diagonal elements ε*_ii_* of the dielectric tensor using Equation 1:

[1]
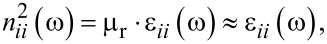


where *i* = *x*, *y*, *z*.

**Figure 3:**
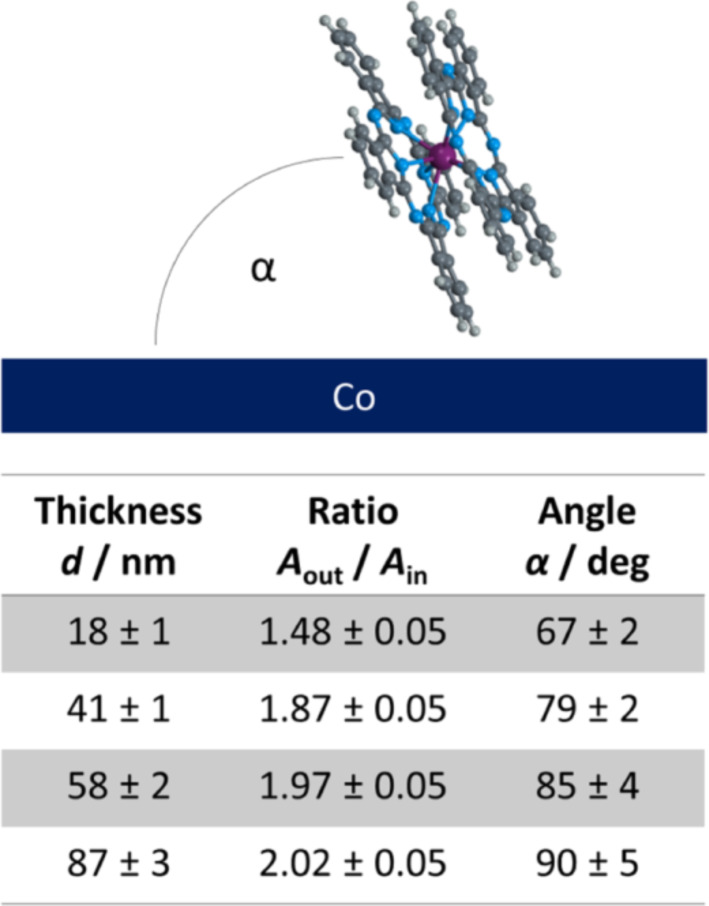
Definition of the molecular tilt angle (top). Average tilt angle of the TbPc_2_ molecules on cobalt (bottom). The thickness of the films was estimated from the ellipsometry data.

From the anisotropy of the extinction coefficient *k* in the Q band region (1.6–2.0 eV), it is possible to estimate the molecular orientation [13–14]. According to the model described in [16] we assume that two electronic transition dipole moments in the Q band are parallel to the ligand plane and that all molecules have the same tilt angle α with respect to the substrate, but with a random azimuthal orientation in the substrate plane. It was shown that under these assumptions the average molecular tilt angle α can be written as [16]:

[2]
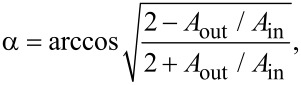


where *A*_in_ and *A*_out_ are the areas under the in-plane and out-of-plane component of the extinction coefficient, respectively (see Figure 2). The resulting average angles between the molecules and the substrate plane can be found in Figure 3. The Sessoli group reported TbPc_2_ molecules which lay in a monolayer evaporated on polycrystalline gold and cobalt as well as standing molecules in a 200 nm thick film by exploiting synchrotron methods [17–18]. Thus, we expect that the first TbPc_2_ layers will similarly lie on the polycrystalline Co films due to their low roughness (rms: 0.4 nm, obtained by AFM measurements). At this point it should be mentioned that the ellipsometry investigations of the molecular orientation in films with smaller thickness is difficult due to the low sensitivity to the out-of-plane component of the optical constants. In particular, this is due to the presence of a metallic layer beneath the organic layer. The molecular tilt angle increases with increasing films thickness, which was also shown for H_2_Pc on PTCDA by utilizing spectroscopic ellipsometry and magneto-optical Kerr effect spectroscopy (MOKE) [16]. According to Equation 2, only values between 0 and 2 are allowed for *A*_out_/*A*_in_. Nevertheless, for the thickest TbPc_2_ layer, the ratio *A*_out_/*A*_in_ slightly exceeds the limit of 2. This could be related to errors in the ellipsometry fit. Therefore, we assume standing molecules for films thicker than 80 nm.

### Topography characteristics

**Figure 4:**
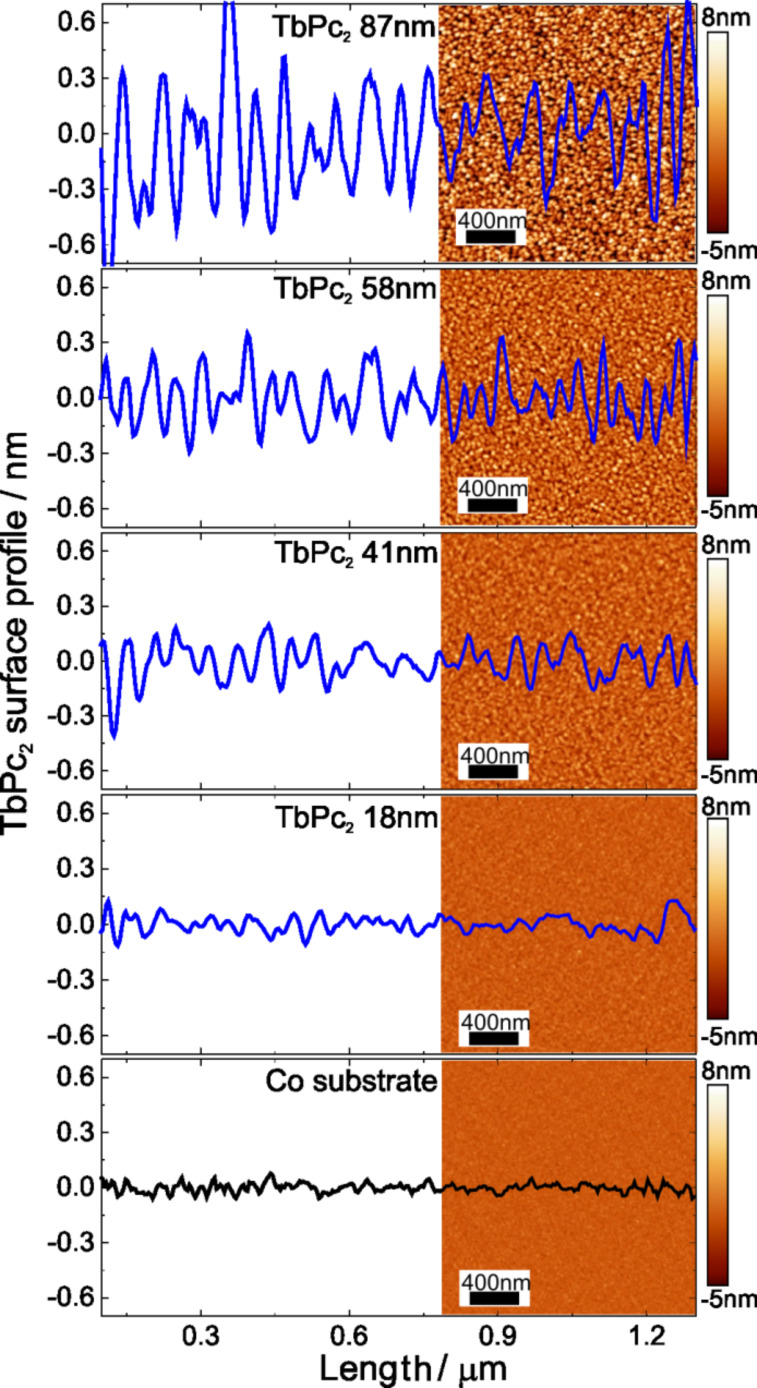
AFM topography characteristics of TbPc_2_ thin films. Line scan profiles and AFM surface images for TbPc_2_ films of 18, 41, 58 and 87 nm deposited on a 25 nm thick Co film.

**Figure 5:**
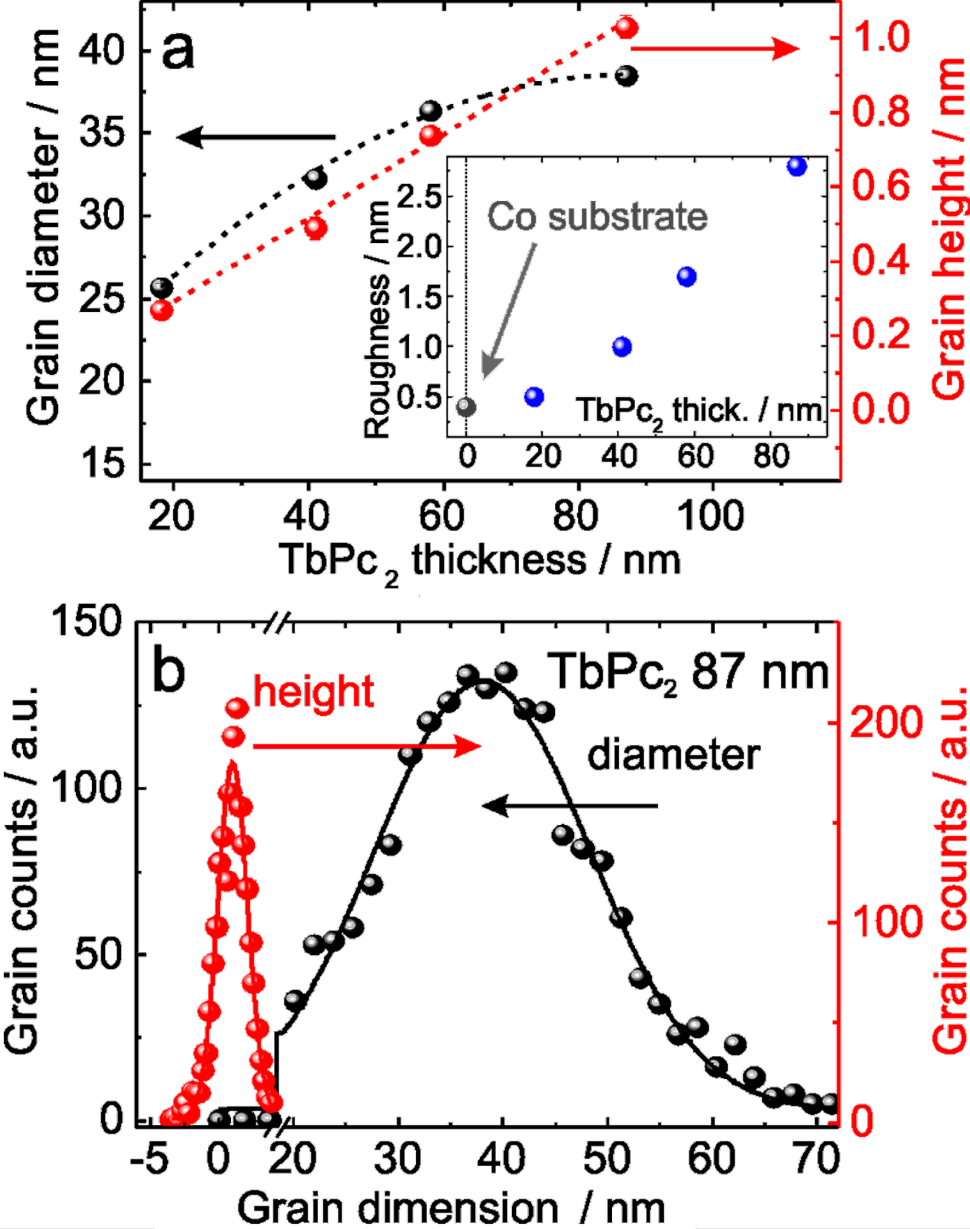
AFM statistical analysis of TbPc_2_ thin films. (a) Average grain diameter and height as a function of the organic film thickness. Dotted lines are guide to the eye to show the tendency of the grain height and grain diameter with the thickness. The inset shows the dependence of the roughness of the organic films thickness. (b) Diameter and height histograms for an 87 nm TbPc_2_ film. Error bars (with sizes comparable to plotted dot symbols) in (a) are obtained from Gaussian fits in histograms as shown in (b).

To investigate the topographic characteristics of the TbPc_2_ thin films on Co substrates, a detailed analysis of the grain size evolution is performed as a function of the organic film thickness. Figure 4 shows AFM images with areas of 2 × 2 µm^2^ for TbPc_2_ films as well as the Co substrates. An increase of the roughness is observed as a function of the thickness of the organic film (see inset in Figure 5a). Scan profiles in Figure 4 show the average grain heights over the sample surface. These profiles also support a clear variation of the topographic characteristics of the TbPc_2_ films as the thickness is increased.

In order to perform a detailed quantification of the TbPc_2_ topographic grain characteristics, a statistical analysis via histograms is used to calculate the average grain diameter and height from the topography images shown in Figure 4. Figure 5a shows that the height of the grains follows a linear increase while the average grain diameter tends to reach a maximum size of about 38–40 nm with increasing organic film thickness, as expected for an unheated substrate during the deposition [19]. As an example of the statistical analysis performed, Figure 5b shows a histogram of the grain height and diameter for the case of the 87 nm TbPc_2_ film. This analysis considers areas of 2 × 2 µm^2^ shown in Figure 4, which contain approximately 2 × 10^3^ grains on the surface. The statistical analysis was performed on different locations of the organic films, revealing similar results. This reinforces the reliability of the data and dismisses the possibility of error during the AFM measurements on a particular location of the sample surface. Further information on the statistical analysis can be found in Supporting Information File 1, Figure S2 and Figure S3.

### Current sensing atomic force microscopy

Conductive atomic force microscopy techniques are well-established methods for local electrical characterization in organic materials [20–24]. In this work, we employ the cs-AFM technique in order to investigate the local transport properties of TbPc_2_ thin films on Co substrates. Due to its high reproducibility and versatility, cs-AFM allows for local current–voltage (I–V) spectroscopy as well as current mapping of particular areas of interest on the organic film. I–V spectroscopy is realized by subsequent sweeping of the applied bias while the AFM conductive probe is located at a fixed location on the sample surface. This procedure is repeated several times over different locations to ensure the reproducibility of the electrical response. On the other hand, in current mapping experiments, a fixed voltage is applied at the probe–sample interface while the probe is scanned over a specific area of the sample surface. These high resolution current maps (512 × 512 points) allow simultaneous information to be obtained regarding topographic and electrical current through the organic material, and establish a direct correlation of the organic topography with electrical characteristics. Also, by repeating current mapping at different locations and with different voltages, we are able to reconstruct the I–V characteristics.

**Figure 6:**
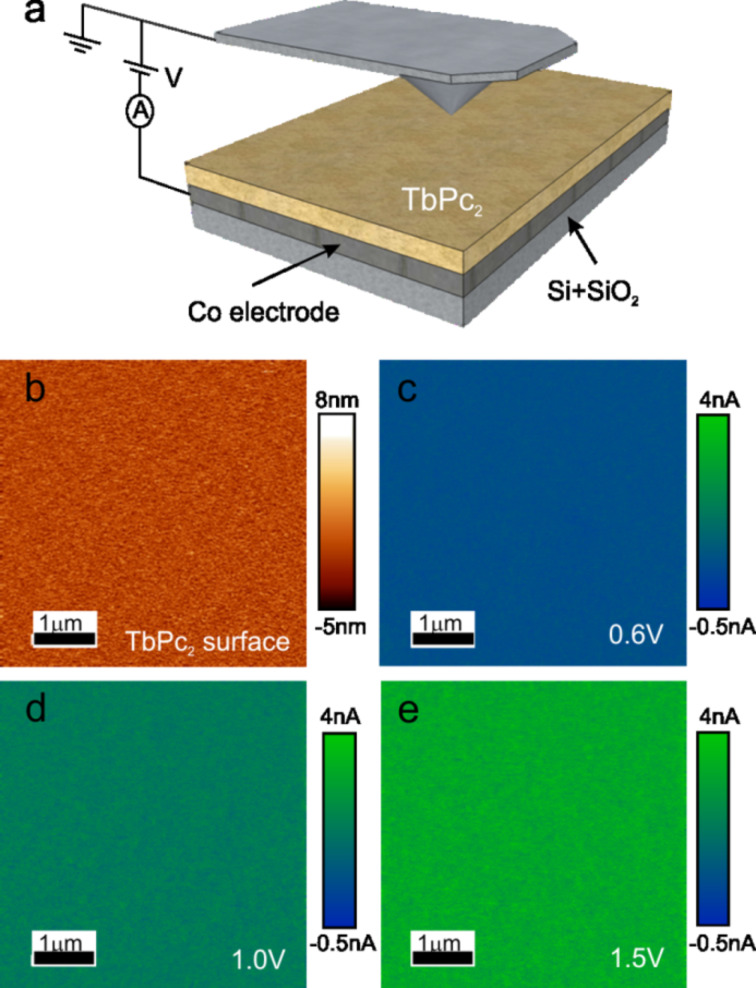
cs-AFM electrical measurements. (a) Electrical setup employed for local electrical measurements via cs-AFM. (b) AFM topography image of an 87 nm thick TbPc_2_ film (5 × 5 µm^2^). Current maps for the same location indicated in (b) for the case of an applied voltage of 0.6 V (c), 1.0 V (d) and 1.5 V (e).

Figure 6a shows a schematic diagram of the set up for local electrical measurements. A conductive AFM probe placed directly in contact with the TbPc_2_ surface plays the role of the top electrode, while the Co bottom film acts as a back electrode. Samples for cs-AFM measurements were deposited on a Si substrate with a top SiO_2_ layer of 1 µm in order to eliminate possible leakage current.

Figure 6b shows a 5 × 5 µm^2^ topography image of an 80 nm thick TbPc_2_ film which has the highest roughness, with respect to Figure 4 and Figure 5. It is worth pointing out that for the case of electrical AFM measurements, special conductive probes with a larger radius compared to the high resolution probes used in the topographic studies (see Experimental section for more details) are utilized. Therefore, a lower topography resolution is expected in topography images acquired during electrical measurements (e.g., Figure 6b). The electrical response of the film, for the same location indicated in Figure 6b, is explored under different applied voltages, as indicated in Figure 6c–e. We observe highly stable and uniformly transport characteristics for all TbPc_2_ film thicknesses investigated. This suggests a uniform distribution of the electrical charge flow through the organic film.

**Figure 7:**
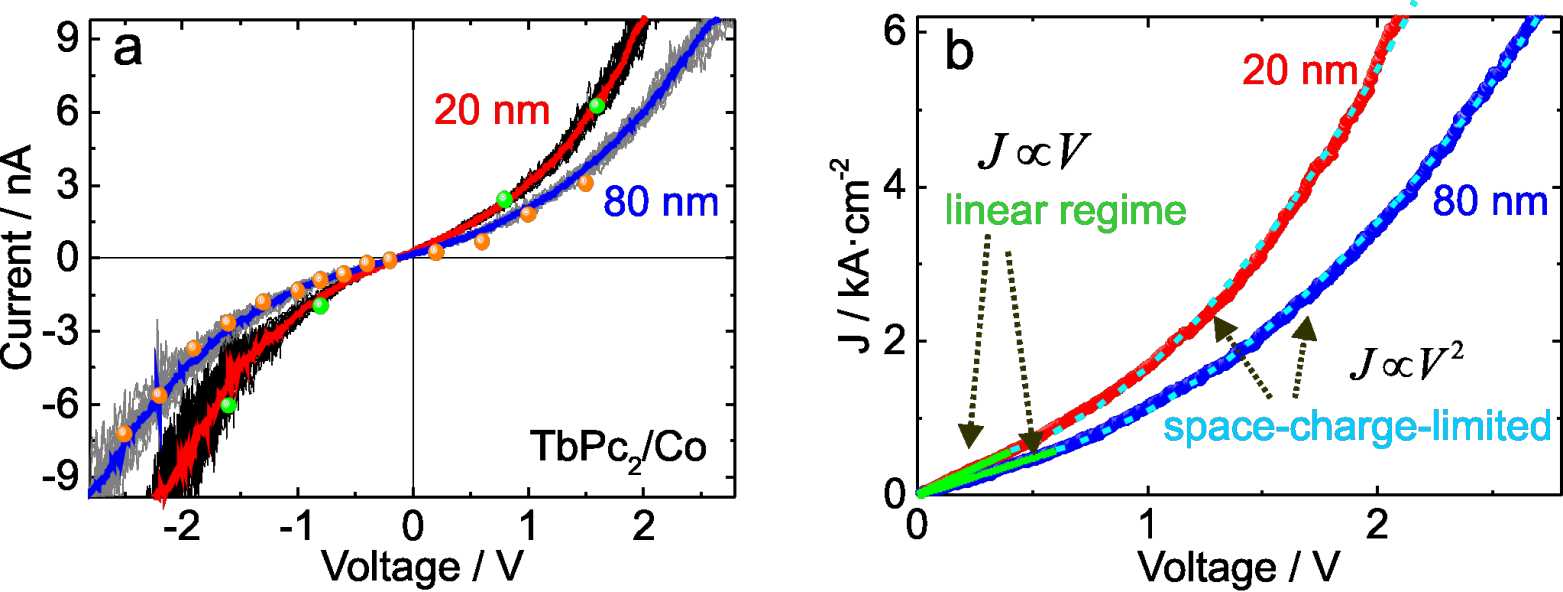
Transport mechanism for TbPc_2_ thin films. Red and blue solid lines indicate the average of 20 local I–V spectroscopy cycles. (a) Current–voltage characteristics for TbPc_2_ thin films. Grey and black data in (a) represent the local I–V spectroscopy cycles. Dotted data represent the current value obtained via current maps. Each dot represents the average of 512 × 512 data points acquired during the AFM scanning. (b) Current density response. Solid green lines and dashed blue lines correspond to the linear and space-charge-limited transport regimes, respectively.

To further investigate the transport mechanism in TbPc_2_ thin films, a series of local I–V spectroscopy measurements on different locations along the organic material were performed. Figure 7a shows the transport response for the case of 20 and 80 nm thick TbPc_2_ films. Here, the AFM probe is fixed at one single point on the surface while the voltage is swept for around 20 consecutive cycles (grey and black areas). Solid lines show the average electrical current. In order to verify the reproducibility of the I–V spectroscopy results, a series of current maps were also obtained at different applied voltages for both organic films. I–V characteristics were then reconstructed by obtaining the average current corresponding to 512 × 512 data points from current maps as the ones shown in Figure 6c–e. Solid dots in Figure 7a correspond to the I–V characteristics reconstructed with current maps and indicate the high reproducibility of the transport measurements for TbPc_2_ organic films performed via cs-AFM.

The current values measured for the TbPc_2_ thin films are close to those reported for the case of CuPc [23] and up to three orders of magnitude higher than those reported for similar organic films investigated via cs-AFM techniques such as metalloporphyrin thin films on Ni substrates [20]. In general, due to their planar structure, phthalocyanines are known to exhibit a higher hole mobility as compared to the porphyrins [25], which could lead to a higher current. When comparing the topographic characteristics of TbPc_2_ samples with 20 nm and 80 nm (see Figures 4 and 5), the variation with grain size would induce more grain boundaries for electron scattering in the thinner samples when compared with the ticker samples. Thus, a different amount of scattering centers might be responsible for the fact that the electric current scales gentler than the expected laws with the film thickness (1/*L*).

Figure 7b shows the current density–voltage characteristics for the TbPc_2_ films. In order to obtain the current density from the cs-AFM measurements, we follow a similar approach as presented by Reid et al. [26], where the contact area between probe and sample is determined assuming a tip indentation of 1 nm for the same kind of Pt-coated hemispherical probes used in this work. This results in a circular contact area with a diameter (*P*_d_) of 14 nm. Here, we ensure that the probe–sample force is kept constant during the electrical measurements and no extra force, which could eventually modify the contact area, is applied.

The electrical response of the TbPc_2_ thin films presents a transition from a linear ohmic-like transport regime for low voltages to a square law dependence for high voltages. These results appear to be in agreement with a space-charge-limited current process (SCLC). According to G. Horowitz et al., the linear current–voltage characteristics can exist in the SCLC model and come from electrons hopping from one insulating state to the next [27]. Hence, the transport regime of TbPc_2_ thin films would follow such transition behavior:

[3]
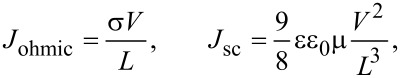


where *J*_ohmic_ and *J*_SC_ are the current densities for each regime, σ is the low voltage conductivity, *L* is the thickness of the organic film, ε is the relative dielectric constant, ε_0_ is the permittivity of free space, µ is the charge carrier mobility and *V* is the applied voltage. From the ohmic-like regime (see Figure 7b) we obtained parameters of σ/*L* equal to 1.38 and 0.97 kA/cm^2^ V for the samples of 20 and 80 nm, respectively. If the mobility is higher along the crystalline direction, where an overlap of the π orbitals of the neighboring molecules exists, then the mobility might be higher perpendicular to the molecular plane as opposed to parallel to it. Assuming the same kind of charge for the carriers and the same charge density per unit volume in all films, a reduction in the ratio σ/*L* corresponds to a reduction in the carrier mobility in the direction perpendicular to the substrate. This is in agreement with the increasingly standing molecules. The validity of the model applied for the ohmic region should be granted in both cases, since the condition is that the amount of injected carriers is low compared to that of thermally generated carriers.

Once the modelling of the current density characteristics for TbPc_2_ thin films is derived from cs-AFM measurements, it is necessary to account for the electrode geometry in the AFM electrical experiments (AFM probe geometry). We apply the modelling approach described in [26], as we use the same AFM probe geometry. Here, the system is treated as an intermediate case between a situation with infinite plane–plane electrodes and a situation with point–plane electrodes. For the electrode-modified electrical characteristics, a semi-empirical expression for the current density determined via cs-AFM is given by [23,26]:

[4]
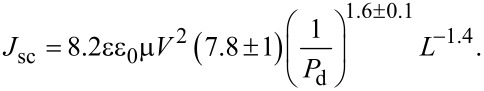


By comparing Equations 3 and 4, one can see that current density measurements via cs-AFM consider a thickness dependence from *L*^−3^ to *L*^−1.4^ for the case of the Pt-coated AFM probes used in this work [26]. Applying this geometry-corrected model to our current density measurements, it is possible to quantify the charge carrier mobility for the TbPc_2_ thin films on a nanometer scale. The values of *L* and *P*_d_ are fixed by the film thickness and tip geometry, respectively. For the dielectric constant we consider a minimum value of ε = 4.5, which is the real part of the dielectric function at the lowest photon energy (1.3 eV) used in our ellipsometry experiment (see Figure 2). As a maximum value for the dielectric constant we used ε = 13, which was previously proposed to describe the hole transport along the phthalocyanine ligand plane in CuPc films [28]. This would be the case if all phthalocyanine molecules were perfectly aligned with their ligand plane perpendicular to the electrodes. From the SCLC fitting in Figure 7b we obtain charge carrier mobility values from 0.80 × 10^−4^ cm^2^ V^−1^ s^−1^ (for ε = 13) to 2.31 × 10^−4^ cm^2^ V^−1^ s^−1^ (for ε = 4.5) for the 20 nm TbPc_2_ film. For the 80 nm thick film, the mobility values range from 0.35 × 10^−4^ cm^2^ V^−1^ s^−1^ (for ε = 13) to 1.01 × 10^−4^ cm^2^ V^−1^ s^−1^ (for ε = 4.5). The variation of the mobility values across the film surface in a scan window of 5 × 5 µm^2^ is below 15% (see Supporting Information File 1, Figure S4). These values are comparable to the hole field effect mobility values determined from OFET measurements by Katoh et al. [10] Noteworthy, the mobility is expected to increase with increasing size of the crystalline grains in the film [29]. A decrease in the hole mobility values with increasing film thickness is therefore at a first glance surprising. However, considering the anisotropic hole transport in the phthalocyanines (with higher mobility in the direction of the π–π stacking in a molecular crystal, i.e., perpendicular to the Pc ligand), a change in the molecular orientation from more lying to more standing molecules with increasing film thickness can be responsible for the decrease in the hole mobility values with increasing film thickness. By taking into account the homogeneous current maps recorded at different voltages (see Figure 6), the excellent agreement between the local I–V characteristics and the average current values obtained via current maps (Figure 7), we conclude that the mobility values estimated above are representative for the area of the respective TbPc_2_ film.

## Conclusion

In this work we present the optical, topographic and electrical properties of TbPc_2_ thin films on cobalt by utilizing ellipsometry and AFM techniques. The ellipsometric studies allowed us to determine the average molecular tilt angle in the TbPc_2_ films and this evaluation revealed an evolution from nearly lying molecules in the first layers to standing molecules in a thick film. The current flow through our organic layers is homogeneous within a standard deviation of about 10%, with lower values at the grain boundaries as compared to the top of the grains (see Figure S5 of Supporting Information File 1). A statistical analysis was conducted to determine the size of the grains and it was shown that the lateral expansion of the grains appears to saturate in films at a thickness higher than 58 nm. The I–V characteristics indicate that the transport through the films with thicknesses of 20 nm and 80 nm is governed by the SCLC regime. By applying a SCLC model adapted for the I–V characteristics obtained from cs-AFM measurements, we estimate the hole mobility in TbPc_2_ films on cobalt substrates to be in the range from 0.35 × 10^−4^ cm^2^ V^−1^ s^−1^ to 2.31 × 10^−4^ cm^2^ V^−1^ s^−1^, depending on the film thickness and the dielectric constant considered. The AFM-based approach implemented here allows important transport properties such as current density homogeneity and the local charge carrier mobility to be quantified. The nanoscale resolution achieved here for the characterization of organic systems such as TbPc_2_ thin films is crucial for future molecular spintronics applications.

## Experimental

### Sample preparation

Cobalt films were grown by electron beam evaporation on a Si(111) substrate covered by a oxide layer with different thickness (VASE, AFM: 1.5 nm and cs-AFM: 1 µm) at a rate of 1.25 nm/min under UHV conditions (10^−8^ mbar). The substrates were cleaned in acetone and ethanol for 5 minutes each in an ultrasonic bath. On top of the cobalt, the TbPc_2_ films were prepared by organic molecular beam deposition at a rate of 0.5 nm/min at a pressure below 10^−7^ mbar. The evaporation took place at a temperature of about 400 °C in the Knudsen cell. The samples were kept at room temperature during all depositions. The preservation of the molecules in a film was checked by UV–vis and Raman spectroscopy.

### Ellipsometry measurements

VASE measurements were performed ex situ with a Woollam T-Solar Ellipsometer in the spectral range of 0.7–5.0 eV with an energy step width of 0.02 eV. Three different angles of incidence (50°, 60° and 70°) were exploited for increased sensitivity of the optical anisotropy of the films. The initially linear polarized light becomes elliptically polarized light after reflection on the sample. The elliptical polarization state is described by the experimentally measured quantities Ψ and Δ, according to

[5]
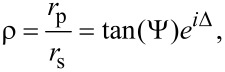


where ρ is the ratio between the Fresnel reflection coefficients for p- and s-polarized light. The ellipsometry data were analyzed using the WVASE 32 software from J. A. Woollam Co. [30].

### AFM measurements

AFM measurements for topography analysis and electrical characterization were performed on an Agilent 5500 AFM system. Measurements were performed under a controlled N_2_ environment to preserve the integrity and avoid exposure of the organic films to ambient conditions. Topography measurements were performed in AC tapping mode, which guarantees minimal contact between the AFM probe and the organic film. Ultra sharp (4–10 nm radius) Olympus cantilevers allowed high sensitivity measurements. cs-AFM measurements were performed in contact mode using special Pt-coated Si cantilevers with a spring constant of 0.2 N/m and typical radii of about 20–25 nm. The voltage is applied directly to the bottom Co electrode. The grounded conductive cantilever is therefore used as a top electrode for local I–V spectroscopy as well as current mapping experiments. AFM topography analysis and current maps images were analyzed using WSxM and Gwyddion software packages [31–32].

## Supporting Information

The Supporting Information shows the raw data obtained from ellipsometry and the corresponding model fit for one sample. Furthermore, a statistical analysis of the AFM data is included.

File 1 Ellipsometry and AFM analysis.
