# Supplementary material for: Optical properties and electrical transport of thin films of terbium(III) bis(phthalocyanine) on cobalt
Source: Beilstein J Nanotechnol. 2014 Nov 11;5:2070–8. doi: 10.3762/bjnano.5.215 (PMC4273272; doi:10.3762/bjnano.5.215)
Supplement: File 1 — Ellipsometry and AFM analysis. [file Beilstein_J_Nanotechnol-05-2070-s001.pdf]

Supporting Information

for

Optical properties and electrical transport of thin films of terbium(III) bis(phthalocyanine) on cobalt

Peter Robaschik[1], Pablo F. Siles[2,3], Daniel Bülz[1], Peter Richter[1], Manuel Monecke[1], Michael Fronk[1], Svetlana Klyatskaya[4], Daniel Grimm[2,3], Oliver G. Schmidt[2,3], Mario Ruben[4,5], Dietrich R. T. Zahn[1], and Georgeta Salvan*[1]

Address: [1]Semiconductor Physics, Technische Universität Chemnitz, Reichenhainer Straße 70, 09107 Chemnitz, Germany, [2]Material Systems for Nanoelectronics, Technische Universität Chemnitz, Reichenhainer Straße 70, 09107 Chemnitz, Germany, [3]Institute for Integrative Nanosciences, IFW Dresden, Helmholtzstraße 20, 01069 Dresden, Germany, [4]Institute of Nanotechnology, Karlsruhe Institute of Technology (KIT), 76344 Eggenstein-Leopoldshafen, Germany, and [5]Université de Strasbourg, Institut de Physique et de Chimie des Materiaux de Strasbourg, CNRS UMP 7504, 23 Rue du Loess, 67034 Strasbourg Cedex 2, France

Email: Georgeta Salvan* - salvan@physik.tu-chemnitz.de

* Corresponding author

Ellipsometry and AFM analysis

Ellipsometry data and modelling

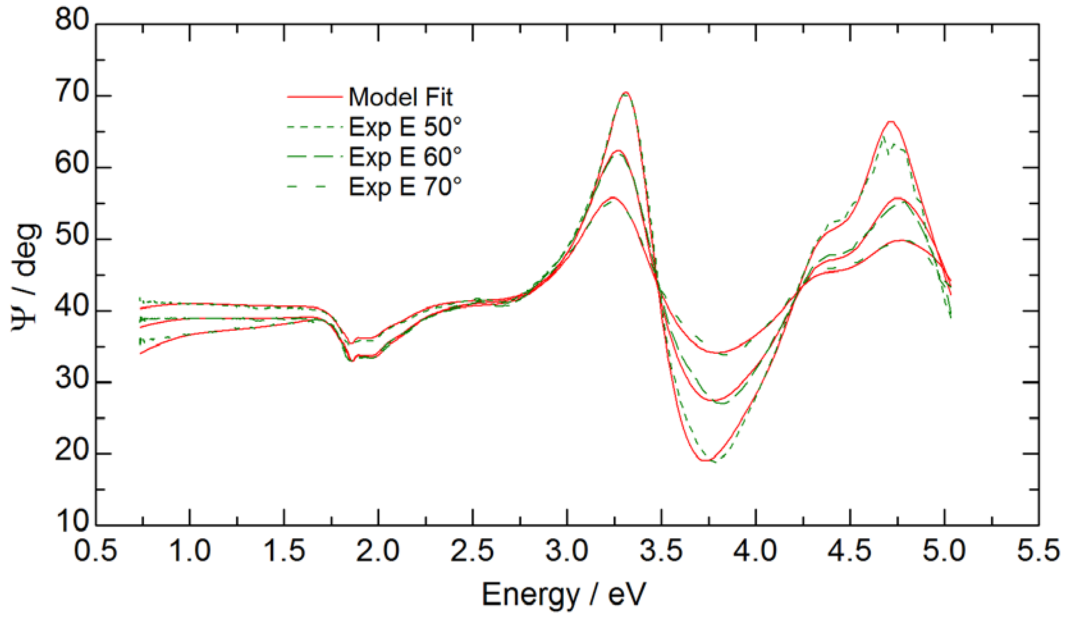

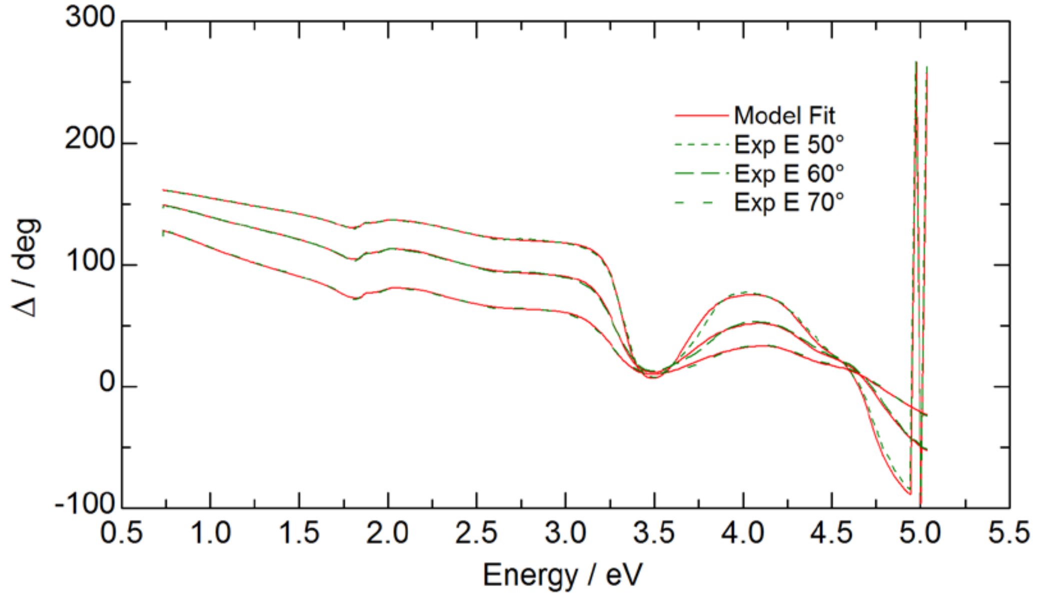

Figure S1: Ellipsometry data of the 41 nm $TbPc_2$ on Co sample (green dashed lines) and the corresponding model fit (red solid lines).

AFM analysis

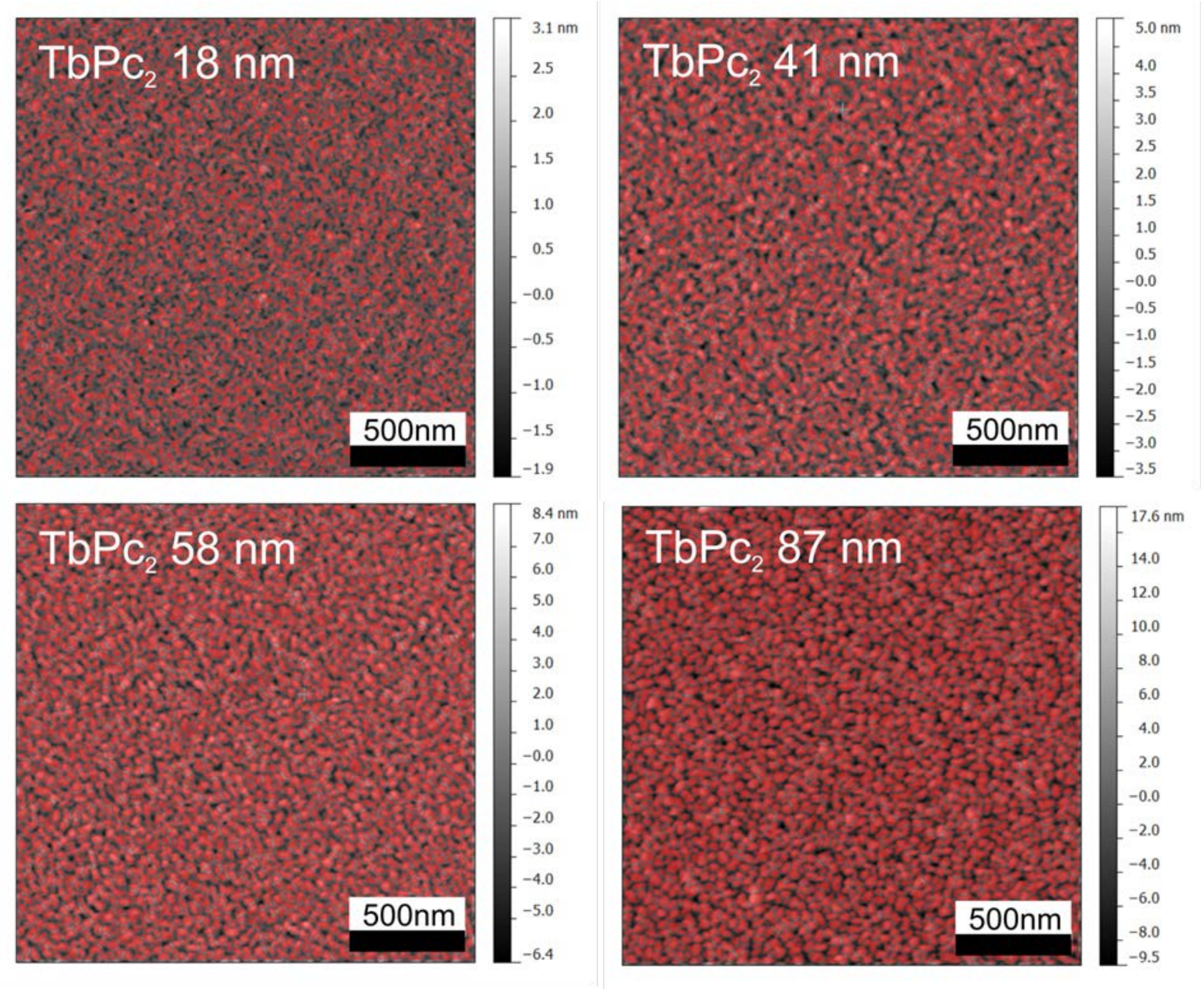

Figure S2: AFM topography images for statistical analysis of $TbPc_2$ grain size evolution as a function of film thickness. Gray-scaled images correspond to the original AFM images shown in Figure 4. Red-colored areas correspond to the analysis grain definition, each red area represent a grain over the organic surface. Statistical analysis allows quantifying parameters such as the number of grains, height and diameter.

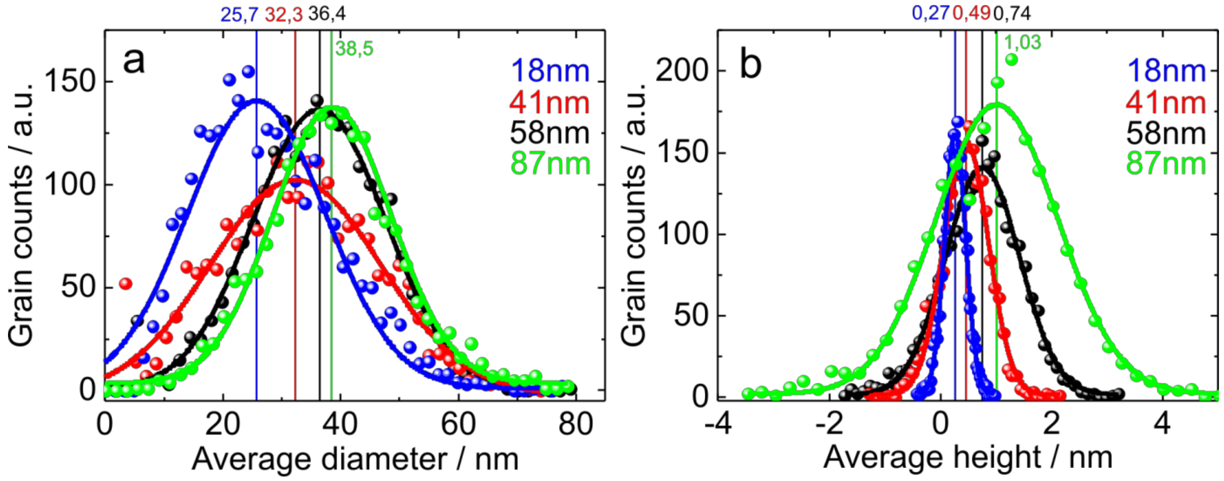

Figure S3: Statistical analysis of grain height and diameter as a function of the thickness of $TbPc_2$. Histograms are obtained from the grain definition shown in Figure S2.

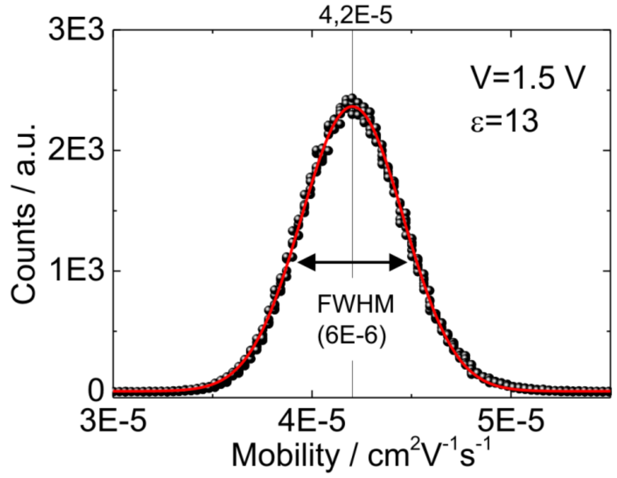

Figure S4: Statistical error estimation for charge carrier mobility given a dielectric constant of 13. Analysis is performed from a current map at 1.5 V. The error is defined as the FWHM value of a Gaussian fit showing the mobility value for all the 512 × 512 data points of the current map.

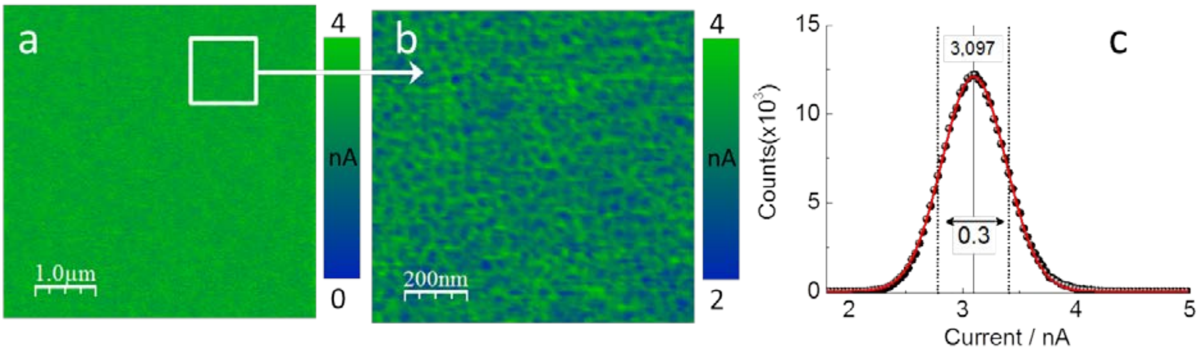

Figure S5: (a) Current map obtained at 1.5 V on an 80 nm thick $TbPc_2$ sample. (b) Higher magnification of current map in (a) for a 1 × 1 μm^2 area as indicated in (a). (c) Electrical current dispersion considering all of the 512 × 512 data points of current map shown in (a).
